# Accurate Blood Flow Measurements: Are Artificial Tracers Necessary?

**DOI:** 10.1371/journal.pone.0045247

**Published:** 2012-09-20

**Authors:** Christian Poelma, Astrid Kloosterman, Beerend P. Hierck, Jerry Westerweel

**Affiliations:** 1 Laboratory for Aero & Hydrodynamics, Delft University of Technology, Delft, The Netherlands; 2 Department of Anatomy & Embryology, Leiden University Medical Center, Leiden, The Netherlands; National Cancer Institute, National Institutes of Health, United States of America

## Abstract

Imaging-based blood flow measurement techniques, such as particle image velocimetry, have become an important tool in cardiovascular research. They provide quantitative information about blood flow, which benefits applications ranging from developmental biology to tumor perfusion studies. Studies using these methods can be classified based on whether they use artificial tracers or red blood cells to visualize the fluid motion. We here present the first direct comparison *in vivo* of both methods. For high magnification cases, the experiments using red blood cells strongly underestimate the flow (up to 50% in the present case), as compared to the tracer results. For medium magnification cases, the results from both methods are indistinguishable as they give the same underestimation of the real velocities (approximately 33%, based on in vitro reference measurements). These results suggest that flow characteristics reported in literature cannot be compared without a careful evaluation of the imaging characteristics. A method to predict the expected flow averaging behavior for a particular facility is presented.

## Introduction

Quantitative *in vivo* blood flow measurements have in recent years provided many new insights in the role of flow-induced forces in the development and functioning of the cardiovascular system [1]–[3]. Examples of application areas include developmental biology [4], the mechanics of platelet activation in thrombus formation [5] and tumour perfusion studies [6].

The progression from qualitative visualisation to quantitative measurements can largely be attributed to the introduction of accurate, robust analysis techniques based on local cross-correlation of image pairs. In particular, microscopic Particle Image Velocimetry (micro-PIV [7], [8]) has become the most widely used tool. In this technique, a camera is used to record the displacement of ‘tracer’ particles in a sequence of images. Using a statistical approach, the local tracer displacements are used to obtain fluid velocities and derived quantities, e.g. flow rate and wall shear stress.

The in vivo blood flow measurements reported in the literature can be divided into two groups: those using artificial tracer particles and those using ‘particles’ that are naturally present (in practice usually red blood cells). Due to the scarcity of experimental data, results from both types of studies are usually directly compared in biological studies, but also used as input for numerical flow simulations. The aim of this study is to see whether such a direct comparison is possible. This is done by performing in vivo measurements using both approaches at the same location in a common model system, the vitelline circulation of a chicken embryo. We show that the approaches can lead to different results, depending on the magnification that is used. In the cases where similar results are obtained here, both approaches underestimate the real velocities. The latter statement is based on in vitro reference measurements using very similar conditions.

### Natural Versus Artificial Tracers

The use of naturally present elements in blood, in general red blood cells (RBCs), as micro-PIV tracers is a natural extension of the classic videomicroscopy visualisation technique. This approach provides a truly non-invasive method to obtain velocities, providing there is optical access. It has been successfully used, among others, in the rat mesentery [9], developing zebrafish heart [1] and embryonic chicken heart and vitelline arteries [10], [11]. Illumination is generally achieved with a continuous light source and high-speed cameras are required to capture the displacement of the RBCs at high magnifications.

The use of artificial tracer particles requires a more complex experimental protocol, as the particles need to be injected [3], [4]. Special care is required in the choice of tracer particles, to ensure that they do not interfere with the organism. Examples of appropriate ‘bio-inert’ particles are liposomes [3] and polystyrene particles coated with polyethylene-glycol (PEG) [12]. Typical sizes are 0.3–1 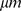 and the particles usually contain fluorescent dye. The latter greatly enhances the image quality, as the scattering of surrounding tissue can effectively be filtered out using band-pass filters. For optimal illumination, pulsed lasers are generally used.

**Figure 1:**
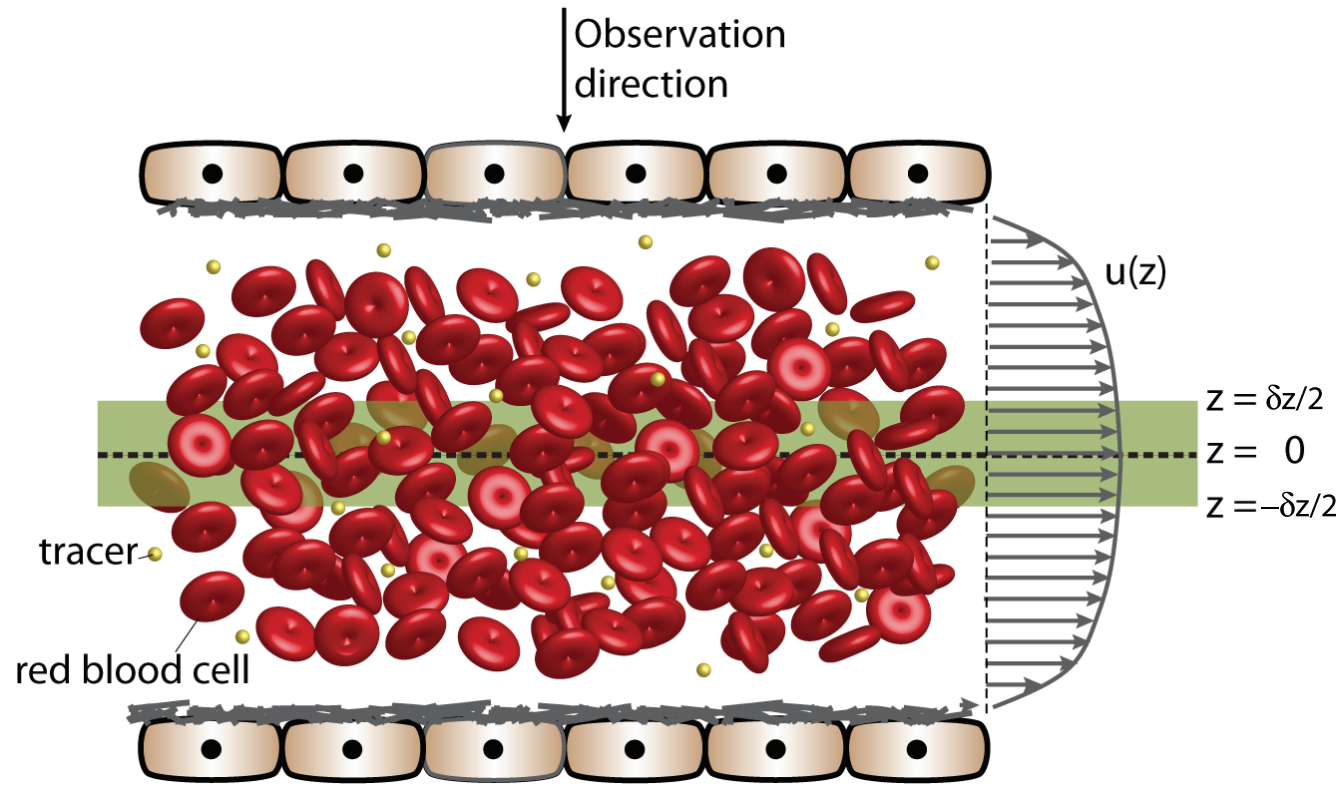
Blood flow and measurement volume. Schematic representation of blood flow measurement using either red blood cells or artificial tracers. The depth of the measurement volume is shown as the shaded region and is bounded by 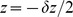 and 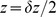.

Due to their relatively small size, these artificial tracer particles can provide velocity information at higher resolutions compared to measurements using red blood cells (approximately 8 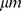 in diameter). In particular near-wall velocity measurements are expected to be more accurate when artificial tracers are used, due to the occurrence of a cell-free layer ([13], see also figure 1). This layer, with a thickness of the order of micrometers [14], can in principle prevent accurate determination of the wall shear stress by means of RBC-based measurements: if there are no red blood cells, no velocities can be determined. Artificial tracers permeate also in this part of the blood vessel [15].

A third possible approach is the fluorescent labeling of formed elements, such as red blood cells [16] or platelets [17]. This approach, which combines aspects of the two main alternatives, is very application-specific and relatively rare compared to the two other approaches.

### Resolution and Measurement Volume

The choice of tracer type has important implications for the effective measurement volume and resolution. Micro-PIV is usually characterized as a method that provides instantaneous measurements of two flow velocity components in a single plane. The field-of-view is determined by the magnification that is used and is typically 100
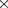
100 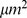 to 1
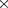
1 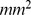. The in-plane resolution that can be achieved is a result of image quality, tracer concentration, camera resolution and the nature of the flow field. It is ultimately determined by the interrogation area size, which represents the size over which cross-correlation is performed in the PIV analysis. The interrogation area size is chosen as small as possible, provided that the analysis gives a good result (e.g. characterized by the number of outliers in the vector field). Usually interrogation areas of 32
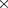
32 or 64
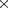
64 pixels are used. In the present study, we can achieve 8.3 and 16.5 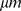 vector spacing (for the medium and high magnification experiments, see later). The actual spatial resolution is twice these values, due to the fact that 50% overlapping interrogation areas are used. Note that this is insufficient to resolve the cell-free layer mentioned in the previous section. The focus of the present study will be on large-scale flow patterns, and not on microscopic near-wall effects.

The out-of-plane dimension of the measurement volume is less straightforward. Unlike conventional PIV, where a thin light sheet determines the measurement volume, the entire volume is illuminated in micro-PIV. This does not mean that all tracer particles contribute equally to the measurement: particles that are in focus will contribute much more than those far away from the focal plane. Images from particles away from the focal plane will be increasingly smeared-out and lower in intensity, so that they eventually can no longer be distinguished from the camera noise. The manner in which particles are focused for a given 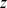 position is determined by the numerical aperture (NA) of the microscope: for high magnifications (with an equivalent high NA), a thin measurement volume can be expected, while lower magnifications lead to thicker volumes. For visualisation, this behavior is conveniently characterized by the depth-of-field of the microscope. However, this depth cannot be interpreted as thickness of the measurement volume. Based on assumptions for the particle image (e.g. a Gaussian intensity distribution) predictions for the effective thickness of a micro-PIV measurement have been derived [18]. This thickness, called ‘depth-of-correlation’ (DOC), is strongly influenced by the tracer particle size. To give an indication of typical DOC values (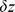), table 1 shows the predictions based on the model by Olsen and Adrian [18] for 1.28 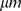 tracer particles and RBCs at two magnifications (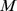 = 12.5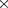 and 25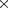; NA = 0.29 and 0.45, respectively). Note how for medium magnification (12.5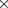), the DOC in the RBC measurements is nearly five times the vector spacing distance (i.e. half the in-plane spatial resolution).

**Table 1:** Overview of imaging characteristics.

	tracer ( 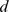 = 1.28 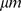 )	red blood cell ( 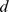 = 8 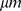 )
M = 12.5×	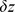 = 28.8 *μm*	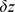 = 74.5 *μm*
M = 25×	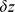 = 11.7 *μm*	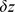 = 42.6 *μm*
M = 12.5×	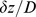 = 0.288	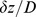 = 0.745
M = 25×	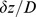 = 0.117	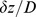 = 0.425
M = 12.5×	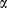 = 0.940	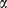 = 0.815
M = 25×	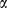 = 0.995	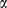 = 0.972

The depth-of-correlation (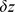) for tracers and red blood cells for the two magnifications used, based on the model by Olsen & Adrian (2000). A typical blood vessel diameter (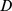) of 100 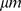 is used to illustrate the relative dimension of the DOC. The values of the ratio of measured and actual centerline velocity (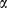) are here based on spatial averaging and a diameter of 100 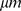.

The DOC indicates to what extent the velocities are averaged in the 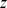 direction, see also figure 1: if the focal plane is aligned with the centerline of a blood vessel (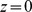), tracer particles within a distance 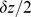 will contribute to the cross-correlation process that is used for the velocity estimate. Unfortunately, the actual averaging process is far from trivial. For relatively thin slices, the measured velocity can be approximated by a spatial average:
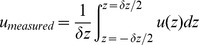
(1)


For simplicity, we here assume that in-plane gradients are small, i.e. the interrogation areas are small compared to the length scales characterising the velocity gradients in 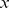 and 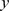 direction. If we assume a parabolic velocity profile, the ratio of the measured velocity and the centerline velocity (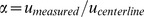) can be calculated [19]. Note that the assumption of a parabolic flow profile is a reasonable first approximation, as the flows under consideration are generally in the laminar (‘Poiseuille’) flow regime [20]; the experimental results described later further illustrate this. For infinitely thin measurement volumes, 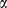 approaches 1, for very thick volumes it reaches a limiting value of 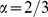. The values of 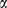 are also shown in the table (assuming a typical diameter of 100 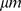). See also the inset in figure S4 in the supplemental material.

The outcome of the cross-correlation and peak detection process used in micro-PIV can no longer be predicted by this simple spatial averaging when the DOC becomes significant compared to the geometry, e.g. the blood vessel diameter [21]–[23]. As can be seen in table 1, the DOC ranges from 11% to 75% of a typical blood vessel diameter of 100 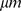. These large DOC values are unavoidable when a large field-of-view is desirable. The difference in DOC for tracer and RBC are the key to understanding the outcome the experiments described in the next section.

Recent work has shown that the DOC can be greatly reduced using confocal imaging, but this also reduces the imaging rate drastically [24], [25]. Alternatively, one can use image preprocessing or sophisticated averaging procedures to reduce the effective DOC [23], [26], [27]. However, these methods have been developed for typical PIV images, which contain relatively sparse small particle images. Physiological hematocrit levels (i.e. volume fraction) lead to overlapping RBC images, so that these methods may not be suitable.

## Methods

The comparison experiments performed here use an extended version of the technique that has been described in detail elsewhere [12], [20]. The extension entails the use of pulsed LEDs to allow for bright-field imaging of the RBCs. Experiments are performed subsequently, not simultaneously, by rapidly changing between the two methods described below.

### Embryo Preparation

**Figure 2:**
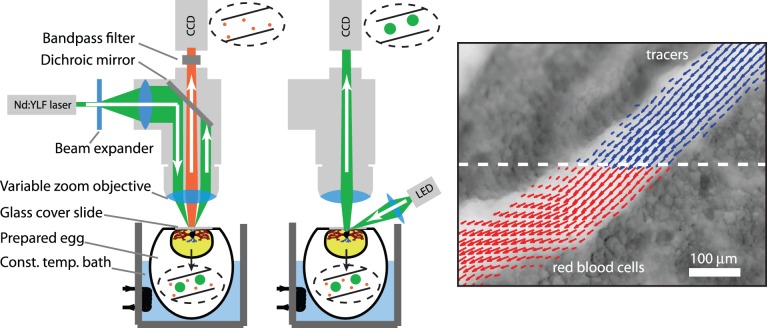
Overview of experimental method. Schematic representation of optical paths for in the vivo flow measurement: *(left)* using fluorescent tracers illuminated by a pulsed laser and *(middle)* using red blood cells illuminated by a pulsed LED. *(right)* A typical result of the measurements: a composite image of the PIV results for the fluorescent tracer particles (top) and red blood cells (bottom).

Fertilized White Leghorn eggs (*Gallus domesticus*) were incubated at 37°C and 60% humidity for three days to reach developmental stages HH17-18. A small window is cut in the shell to allow optical access. After staging and inspection for abnormalities, the embryo is partially submerged in a temperature-controlled bath (37°C), see figure 2. Tracer particles (1.28 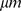 PEG-coated polystyrene particles containing Rhodamine B; Microparticles GmbH) are injected in a vitelline artery using a 10-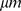 needle and a microinjector. This injection is done using the stereo objective of a combined mono/stereo epifluorescent microscope (Leica MZ 16 FA). After successful injection, the embryo is covered with a microscope slide to prevent desiccation and minimize optical distortion. Measurements are performed in the larger vessels of the extra-embryonic circulation (vitelline arteries and veins). At this developmental stage, these form a two-dimensional network that floats on the yolk.

### Artificial Tracer Measurements

The left-hand side of figure 2 shows the configuration that is used for the micro-PIV measurements using artificial tracers. The expanded beam of a pulsed double-cavity Nd:YLF laser (New Wave Pegasus, 10 mJ/pulse at 527 nm) is used to illuminate the tracer particles through the objective. The fluorescent light is collected by the same objective, but can pass through the dichroic mirror, so that it can be captured on a CCD camera (PCO Sensicam QE, 1376
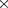
1040 pixels; 2
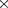
2 binning). Synchronisation of all hardware and data storage is done on a PC running DaVIS 7.1 (LaVision GmbH). Typically, 500 image pairs are recorded at a frame rate of 9.9 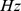, with a laser pulse delay time 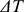 = 5000 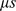. This delay time results in a *maximum* tracer displacement of 8–10 pixels between the two frames of an image pair, which was found to be optimal for PIV analysis. The images of this measurement only contain the signal of the artificial tracer particles.

#### Red blood cell measurements

Figure 2 (*middle*) shows the modified configuration that is used for the measurements using red blood cells. The laser cannot be used for bright-field illumination, as the coherence of the light leads to interference patterns in the image. Continuous white light sources (e.g. arc lamps) cannot be used in combination with the CCD cameras in ‘double-frame’ mode, as the (fixed) exposure time of the second frame is orders of magnitude longer than the first, resulting in an overexposed image. Using the camera in the conventional single-frame mode, so that the exposure can be controlled, severely limits the maximum velocities that can be measured - the delay time between frames is in this case equal to the inverse of the maximum frame rate of 19 Hz (in single-image mode). An alternative is the use of a high-speed camera [10], but this limits the measurement time to a few cardiac cycles and significantly reduces the signal-to-noise ratio of the fluorescence images. Recently available high-intensity pulsed LEDs overcome the limitations of both laser and arc lamps, as they provide pulsed, yet incoherent light [28]. The drawback is that the light cannot be collimated efficiently, so that they cannot be used for illumination through the objective (most of the light is lost inside the microscope). Therefore, the LED is here used to illuminate the field-of-view directly, from the side of the microscope (figure 2, *middle*).

For the red blood cell measurements, the dichroic mirror and the optical filter are removed, so that the camera captures the bright field image. Removal of the mirror and filter did not introduce any noticeable shifts in the images in tests with calibration targets. The images of these measurements contain scattered light (see e.g. figure 2 (*right*), which shows an average image as background), the red blood cells appear as black discs in the individual frames. Fluorescent tracer particles could not be observed, due to their very low intensity. All hardware and settings are identical to the ones used for the artificial tracer measurements.

#### Protocol and data analysis

Blood vessels are selected in a range that is feasible for both type of measurements: blood vessel that are too large (over 200 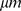) lead to a blurred image due to the long optical path through blood, in particular in the RBC images. Zooming in on smaller vessels (smaller than 50 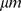) results in very low artificial tracer number densities in the images. The present amount of injected tracer suspension is chosen so that the cardiovascular system is not affected [20]. Injection of larger amounts of particle suspension will increase the preload and thus functioning of the heart, so that the measurements are no longer non-intrusive. Therefore, blood vessels in the range of 75–150 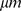 are chosen. In particular, we select regions with an easily identifiable structure (e.g. a bifurcation), so that the overlap between subsequent measurements can easily be verified. The focal plane is placed at the approximate centerline of the blood vessel(s). Recordings are performed subsequently, either starting with the RBC measurements or with the artificial tracer measurements. Recording of one type of tracer typically takes 1 minute, changing the filters and adjusting the triggering of the illumination is achieved in 20–30 seconds. After completion of both experiments, a check is performed using the original setting to see if the same field of view is imaged and the focal plane is still correct.

The PIV image pairs are first corrected for drift. This is done by cross-correlating the images (in part) with a reference image. The images are not phase-locked with the cardiac cycle. Therefore, the phase is first estimated for each frame by obtaining the mean velocity in the entire image. From this velocity signal, the location of each frame within the cardiac cycle can be found by interpolating between the two nearest systolic peaks [20]. The heart rate also follows from this analysis. Only data sets are analyzed where both measurements gave a constant and similar heart rate (at least within 5%).

The image data set is sorted into ten phases of the cardiac cycle. Data within each phase group is processed using an in-house iterative correlation-averaging PIV algorithm [20]: intermediate correlation results are averaged, rather than vector fields to improve the signal-to-noise ration [7]. The final stage of the analysis uses 32
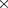
32 pixels interrogation areas with 50% overlap, corresponding to 8.3 and 16.5 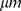 vector spacing distance for the two magnifications used (see later). The PIV analysis results in ten vector fields that describe the phase-averaged flow field throughout the cardiac cycle. An example of such a result is shown in figure 2 (*right*). This image is a composite showing the velocity field at systole using red blood cells (bottom half) and using artificial tracers (top half). The background is the average bright-field image to indicate the geometry. Although the data has been matched during the drift correction, an additional check is performed to check the vector field overlap. This is done by calculating the sum of the squared differences between the vector fields of each method for a range of horizontal and vertical shifts. The optimal overlap corresponds to the lowest sum of squared differences between RBC and artificial tracer result. This generally leads to a minor correction that is smaller than the interrogation area size.

Recordings are obtained at magnifications (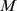) of 12.5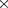 and 25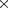, which are, as will be shown later, exemplary for two distinct regimes. These magnifications are also representative of those used in the studies mentioned in the introduction. Several embryos are recorded at each magnification, yet only data of one embryo at each magnification will be presented in a direct comparison as a qualitative illustration of the observed effects; other experiments gave similar results. The outcome of additional experiments using other embryos for the measurements in section *‘Implications for 3D reconstructions’* also support the outcome of the direct comparison.

By translating the focal plane using the automated 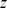-stage of the microscope, a stack of recordings can be obtained that cover the entire volume containing the blood vessels. This way, three-dimensional velocity fields can be obtained (see section *‘Implications for 3D reconstructions’*). The scanning measurements require a significantly longer measurement time (20–30 times longer than a single plane). It was not possible to perform a repeated measurement with the other method, so measurements of different embryos are shown in that section.

#### Medium magnification results

As the results are expected to be dependent on the magnification, we present them in two separate sections. In this section, the results at 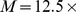 are shown, which we will also refer to as ‘medium’ magnification.

**Figure 3:**
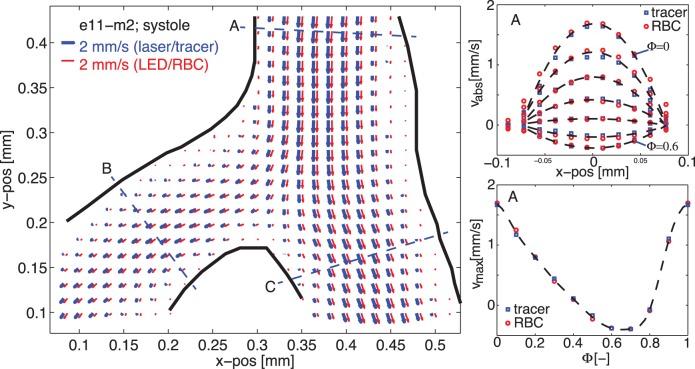
Results obtained at 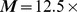. *(Left)*: Superposition of the vector fields using fluorescent tracers (bold, blue vectors) and red blood cells (red vectors) at systole; a small off-set is used between the vector fields for clarity. *(Top right)*: velocity magnitude for both methods along profile A for 7 subsequent cardiac phases, 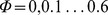; artificial tracer (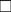) and RBCs (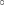). *(Bottom right)*: Centerline velocity at profile A during the cardiac cycle for both methods.

Figure 3 shows the results for a single embryo. The left hand figure shows two superimposed vector fields, representing the velocity field at systole (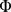 = 0) obtained using artificial tracers and RBCs, respectively. A small offset is used for clarity. The solid curves indicate the wall locations, which were determined by finding the locations where the velocity field approaches zero; this was done using a local polynomial fit. The top right sub-figure shows the velocity along a profile at position marked ‘A’ in the vector fields. It shows the data for the first seven out of the ten phases that make up the cardiac cycle (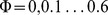). Again, data for both artificial tracer (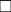) and RBCs (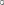) are shown. The dashed lines indicate a parabolic fit for each of the phases (a single fit is used for both data sets for each phase, as it merely serves to indicate the general flow profile). The bottom right sub-figure shows the centerline velocity at profile ‘A’ during the cardiac cycle. The values are obtained from the parabolic fits shown in the top-right figure. The dashed line is a spline to indicate the overall shape. The standard deviation of the differences between artificial tracer and RBC results at the centerline of profile A is 0.04 mm/s (2.4% of the maximum).

Note that the velocity profiles shown in figure 3 are close to parabolic and are not blunted, as is often reported in literature. This can be explained by the low Womersley number of the flow that is studied: 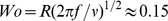, using a radius 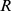 of 75 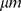, a frequency 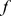 of 2 Hz and a kinematic viscosity of 3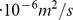. A value of 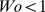 indicates that viscous forces dominate over transient inertia, so that no blunting will be observed [29]. A second cause of blunted profiles is the shear-thinning behavior of blood [30]. Note that we are here working with avian blood, which contains nearly spherical RBCs. These are less prone to form rouleaux than human biconcave-shaped RBCs. This reduces the shear-thinning behavior somewhat [31]. Note that the use of a parabolic fit to describe the results here is a pragmatic choice: it is the simplest shape that appears to describe the data well. By no means does it represent a restriction of the flow measurement technique. More complex profiles can be measured without any problems.

**Figure 4:**
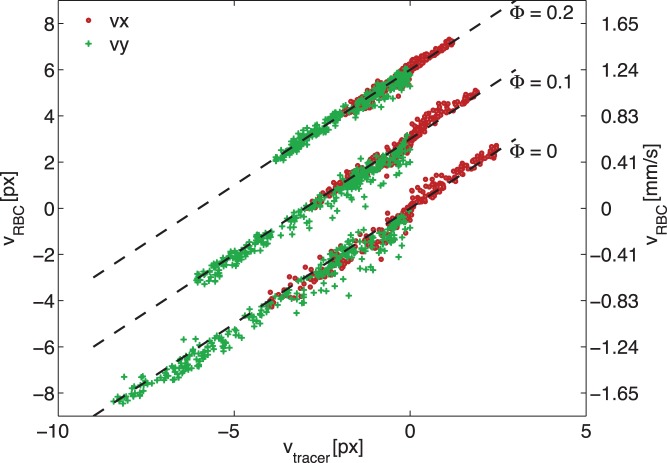
Agreement at 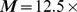. A scatterplot of the displacement measured using red blood cells versus the results using artificial tracers. Three time steps are shown, with an offset for clarity (0, 3 and 6 pixels). Horizontal and vertical components are shown separately ( and +, respectively). The dashed lines indicate perfect agreement.

As can be seen from figure 3, the results from experiments at M = 12.5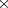 using artificial tracers and RBCs are very similar. For a more quantitative comparison, figure 4 shows a scatterplot of the results. In this figure, the displacement based on RBCs at a given location is shown versus the artificial tracer displacement. Note that the primary axis shows the displacement (in pixels), while the right-hand side axis shows the corresponding velocities (i.e. after multiplication by the scaling constant and dividing by 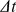). Data for three consecutive phases are shown (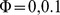 and 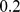), each with a vertical offset for clarity (offset of 0, 3 and 6 pixels, respectively). Horizontal and vertical components are shown as dots () and plus-signs (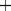). The dashed lines indicate perfect agreement, 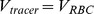.

To characterize the agreement of the results, a linear regression is performed for the results at 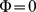. Expressed as a linear function 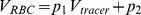, the fit parameters and the 95% confidence intervals are 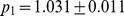 and 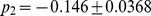 (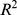 value of 0.983). The standard deviation of the difference between the displacement results is 0.372 pixels (4.4% of the maximum value). This is likely due to random measurement errors in the individual experiments, which would then be 3.1% if they are of equal magnitude (i.e. both RBC and tracer measurement have an independent, random measurement error of 3.1%).

As an additional check, we can verify the data ‘internally’ by checking the conservation of mass of the flow: the sum of the flow rates (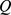) through profiles B and C should match the flow through A. This method has previously been used to characterize the accuracy of flow measurements, in particular for in vivo applications where it is not feasible to obtain reference measurements [32]. Flow rates are determined assuming circular vessels; the diameter and centerline velocity follow from a parabolic fit. For the RBCs, we find 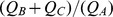 = 0.893 at systole; for artificial tracers we find 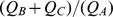 = 0.910. This indicates that both are in reasonably good agreement. A possible explanation for the ‘loss’ of flow might be the fact that the measurement plane is not completely aligned with the actual centerlines of the blood vessels (e.g. the measurement plane might be inclined or one of the blood vessels has curvature in the 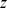 direction).

#### High magnification results

**Figure 5:**
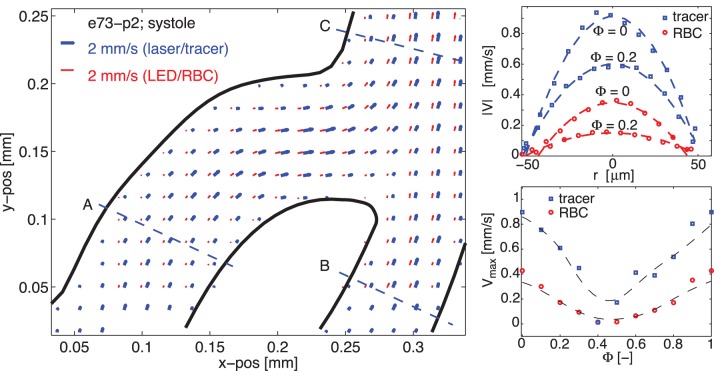
Results obtained at 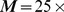. *(Left)*: Superposition of the vector fields using fluorescent tracers (bold, blue vectors) and red blood cells (red vectors) at systole; a small off-set is used between the vector fields for clarity. *(Top right)*: velocity magnitude for both methods along profile A for two time steps (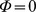 and 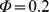). *(Bottom right)*: Centerline velocity at profile A for during the cardiac cycle for both methods.

In figure 5, an overview of the measurement results is shown for 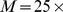, in a similar manner as before. For clarity, only every other vector is shown in the left hand vector field (i.e. 25% of the actual resolution). For the same reason, the upper right plot only gives two velocity profiles at location A. In the bottom right figure, individual splines are shown to indicate the trend of each of the experiments.

**Figure 6:**
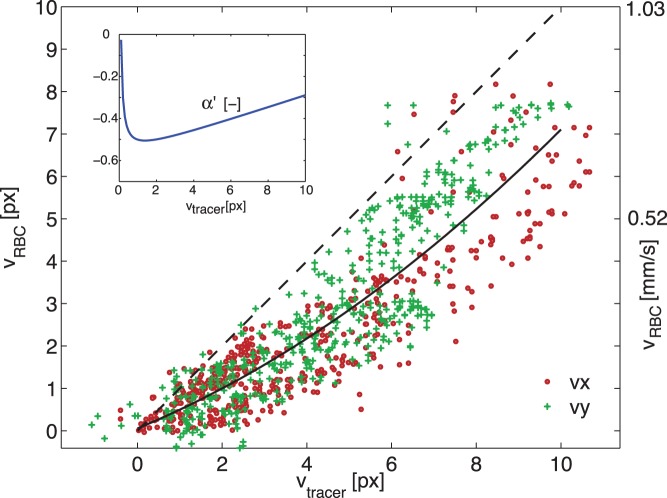
Agreement at 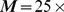. A scatterplot of the displacement measured using red blood cells versus the results using tracers. Data of the first three time steps are shown in one figure; horizontal and vertical components are shown separately ( and +, respectively). The dashed line indicates perfect agreement, the solid line is a quadratic fit. The inset shows the relative underestimation 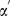 based on the polynomial fit.

As can be seen in figure 5, there is a considerable discrepancy between the two measurements at 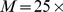: the RBC velocities are systematically lower than the artificial tracer velocities. At the centerline of profile A, there is a 53% underestimation at systole, while the average underestimation during the cardiac cycle is 25%. Figure 6 shows a scatterplot of the first three phases (without offset). As can be seen, there is a systematic underestimation, approaching a difference of 3 pixels for a displacement of 10 pixels.

The standard deviation of the difference between the two data sets is 1.22 px (0.126 mm/s, 14% of the maximum). In the scatterplot we also add a second order polynomial fit (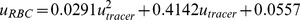, 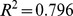 ), which appears to describe the general trend. The root mean square of the deviation of the data with respect to this fit is 0.902 pixels (0.093 mm/s, 10% of the maximum). The inset of the graph shows the relative difference 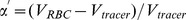, i.e. the normalized difference between the dashed line and the polynomial fit. Note that this parameter 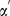 expresses the difference between RBC and artificial tracer results, not the difference with respect to the true centerline velocities (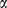).

The balance of flow rates in the bifurcation gives 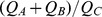 = 1.106 for the artificial tracers and 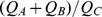 = 0.673 for the RBCs. At location C approximately a quarter of the velocity profile on the right-hand side is missing, due to a low signal-to-noise ratio in that part of the image. Nevertheless, the centerline velocity is captured and the diameter can be obtained from the polynomial fit.

**Figure 7:**
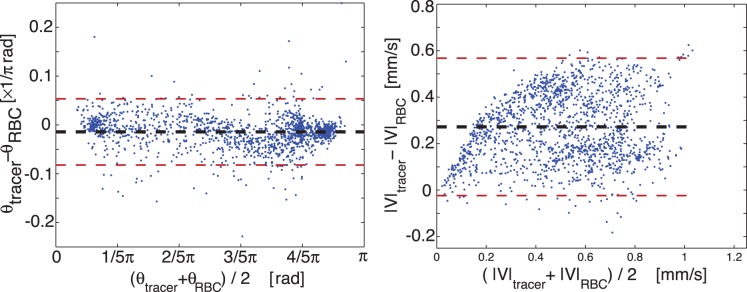
Origin of discrepancy. Bland-Altman plots of the angle (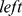) and magnitude (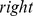) of the measurements using laser 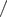 tracers and led 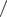 red blood cells. 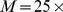. The error in angle is relatively small compared to the error in magnitude.

An alternative approach to characterize the agreement between the results is by making use of Bland-Altman plots for the direction and magnitude of the velocity vectors. These plots, which show discrepancies as a function of the magnitude of a certain property, are shown in figure 7. The horizontal dashed lines show the 95% limits of agreement, corresponding to 1.96 times the standard deviation of the discrepancies. Figure 7 (*left*) shows that the *direction* of both artificial tracer and RBC vectors is very similar (a standard deviation of 6.1). However, the magnitude shows major discrepancies (figure 7, *right*); the standard deviation of local magnitude difference is 0.1478 mm/s, but this value does not take the trend into account. An important secondary conclusion of these Bland-Altman plots is that the directions of the vectors match well, which suggest that the errors are not caused by misalignment of the velocity fields.

## Discussion

For medium magnifications (M = 12.5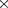), the results agree very well: the velocity fields obtained by red blood cells and artificial tracers are near identical, the small differences can be attributed to measurement uncertainty. This is contrary to what would be expected based on a simple spatial averaging model, see e.g. the values for 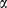 in table 1, which suggest that RBC velocities would be 15% smaller than the artificial tracer data.

At higher magnifications (M = 25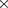), the results of red blood cells and artificial tracers are clearly different. While the local direction of the velocity vectors matches very well, the RBC velocity magnitude appears to be significantly lower than the artificial tracer based results. The relative underestimation (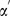) can be up to 50% for displacements of 1.5 pixels. Again, the spatial averaging model is contradicted, as this predicts that both results would agree within the experimental error margins (table 1).

To understand the differences in behavior at the two magnifications, a new synthetic PIV model is introduced. This model serves as a framework in which the experimental results can be interpreted, hence we have chosen to introduce it here rather than in the Results section. The details are given in Appendix S1, we here only summarize the basic concepts. In the model, the process of PIV measurement is mimicked ‘in silico’, so that the influence of measurement parameters on the final result can be studied. The input for the model is a series of particle images, obtained at different 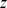 locations (this provides both in-focus and out-of-focus particle images). Using these particle images, the cross-correlation process at each 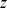 location can be modeled, using a known velocity field. The contributions from each 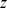 plane are then summed, so that the ‘observed’ correlation function is obtained. The location of the maximum in the latter result is then the displacement that would be obtained from a PIV measurement. The model accurately predicts underestimation observed in reference in vitro measurements [22]. These reference measurements were performed under very similar measurement conditions (same microscope, camera and tracers) in a glass capillary of a diameter comparable to the blood vessel dimensions. The flow in the capillary was driven by a calibrated syringe pump, so that reference flow rate information was available. In this manner, the underestimation of the PIV results could be determined. These results are captured by the new model. As the present experiments are taken under the same conditions, the model can provide the value for 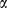 as best estimate in absence of ‘true’ in vivo measurement data.

**Figure 8:**
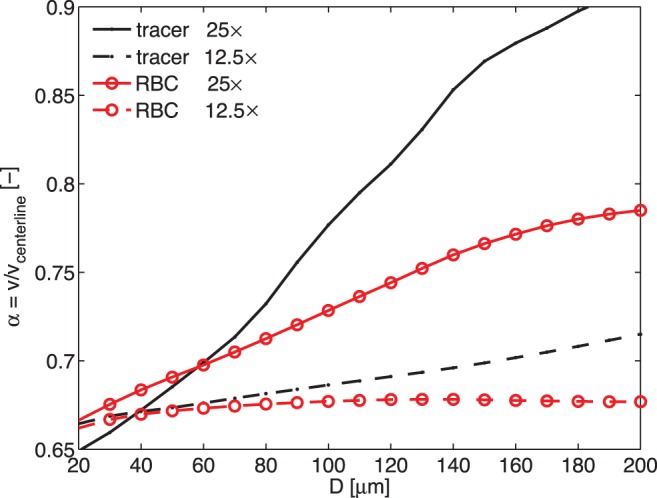
Model prediction for underestimation. Predicted underestimation of the measured velocity (compared to the true centerline velocity) as a function of blood vessel diameter, based on the ‘in silico’ PIV model.

The results for the underestimation (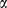) for both tracer types at both magnifications are shown in figure 8. For medium magnification (M = 12.5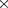 and lower), the underestimation is nearly independent of the vessel diameter and both tracer types will experience an underestimation close to the limiting value of 2/3. At these magnifications, the DOC is large compared to the blood vessel, so that the spatial averaging will approach a limit. Closely related to the large DOC is the observation that the particle images are nearly constant for all 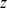 positions within the flow. We here coin the term ‘depth-saturated’ to describe this measurement regime, which implies that a further increase in DOC (viz. a lower magnification) will not lead to further underestimation of the flow.

The model predictions are in agreement with the observations in the *‘Medium magnification results’* section: both methods give the same result, as they both underestimate the centerline velocity by the same, limiting amount. This limiting value of underestimation is similar to what Baker and Wayland observed for the classic dual-slit cross-correlation method for blood flow measurement [33]. They found a correction factor of 1.6 for the mean velocity, corresponding to a value of 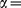 0.8 for the centerline velocity in the present terminology (as the centerline is twice the mean velocity, assuming Poiseuille flow). Note that Baker et al. assume spatial averaging (equation 1, see also [19]), which is not appropriate due to the non-linear averaging behaviour of the PIV technique [34]). Furthermore, there is no influence of the vessel diameter in the Baker and Wayland model.

For higher magnification (e.g. 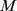 = 25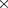), there is a more pronounced difference between the velocities obtained with the two tracer types: for artificial tracers, the underestimation is smaller. This is in agreement with the observations of section *‘High magnification results’*. Note that again both methods are underestimating the true centerline velocities, yet the artificial tracer results are underestimated less. Both are influenced by the vessel diameter at this magnification.

The model described in Appendix S1 assumes sparse images, with non-overlapping tracer images. In reality, especially in the case of red blood cells, this is an oversimplification. At the present developmental stage, the hematocrit is 15–20% [31]. Such a dense suspension of red blood cells will blur the image with increasing optical path length [3]. One can therefore expect that regions close to the objective will contribute somewhat more than regions further away. This may bias the result to the near-wall velocities, leading to a further underestimation. An additional complication - absent in the model - is the possible contribution of tissue outside the blood vessel. If signal of non-moving tissue is also captured in the image, this may further attenuate the velocity result (when using fluorescent tracers, this effect can be assumed to be negligible). These effects can lead to an underestimation that is more dramatic than what the theoretical limit of 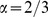 predicts (see e.g. the 50% underestimation in figure 5).

**Figure 9:**
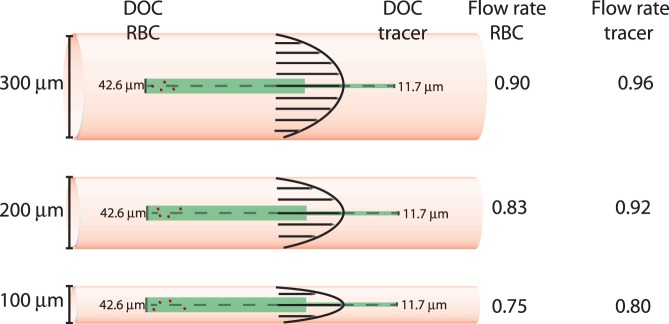
Example of predicted underestimation for various diameters. Schematic representation of depth-of-correlation and resulting flow rate underestimation 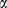 for red blood cells (RBC) and artificial tracers for measurements in geometries of 300, 200 and 100 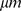. Model predictions for 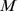 = 25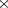.

Apart from explaining the observed differences at the two magnifications, the model provides another important result: for high magnifications, the underestimation is influenced by the blood vessel diameter that is studied. To illustrate this, figure 9 shows the effect of flow rate underestimation for three blood vessel diameters at the same magnification (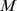 = 25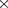) - note that centerline velocity and flow rate are similarly underestimated. This effect is especially relevant when studying, for instance, the embryonic chicken heart. During the cardiac cycle, the diameter of the tube-like heart segments varies significantly [35]. This means that the relative underestimation also varies, so that flow rate measurements need to be corrected differently at each instance. The same phenomenon also becomes relevant when studying bifurcating blood vessels with different blood diameters. The different amounts of underestimation will lead to an imbalance of the flow rates. For instance, if a large (300 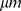) blood vessel branches into a two daughter vessels of 200 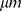 and 100 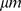 diameter, the observed sum of flows through these daughter vessels will be 8% to 20% lower than the flow through the main branch, depending on the flow distribution ratio. Note that in the present study the variations in blood vessel diameters are relatively small (e.g. figures 3 and 5).

### Implications for 3D Reconstructions

The blood vessels studied so far are more or less confined to a plane, so that a single measurement at the center-plane provides all information needed to describe the flow (assuming blood vessels with a circular cross-section). For more complex geometries, it is desirable to obtain three-dimensional velocity fields. A convenient way to obtain these is by stacking a series of measurements, e.g. by means of a computer-controlled translation stage [12], [20], [24]. We here show that also in this case the choice of tracer material can have a significant influence on the outcome of such a measurement.

**Figure 10:**
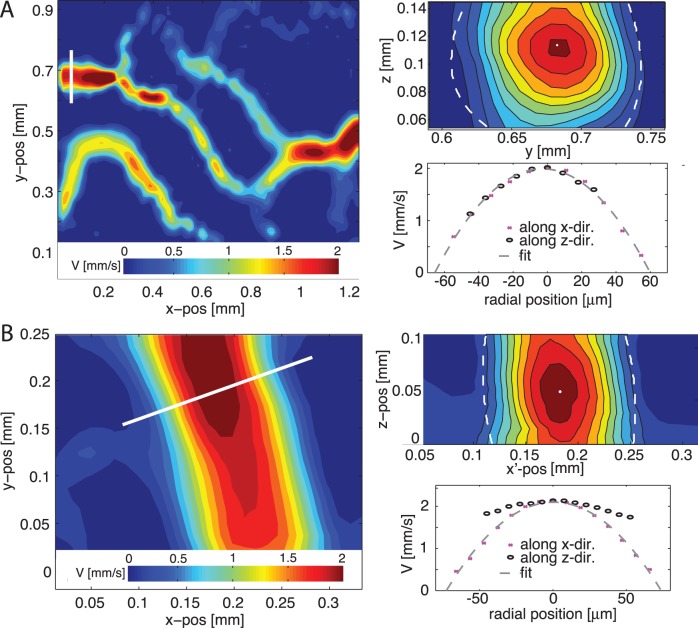
Effect of tracer choice on 3D reconstructions. (A): Three-dimensional reconstruction using tracers. *Left*: single measurement result of the velocity magnitude. *Top right*: cross-section taken along the white line in the left-hand figure. *Bottom right*: in-plane and out-of-plane velocity profile. (B): Three-dimensional reconstruction using RBCs. *Left*: single measurement result of the velocity magnitude. *Top right*: cross-section taken along the white line in the left-hand figure; 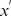 is the axis in the coordinate system aligned with the profile. *Bottom right*: in-plane and out-of-plane velocity profile. 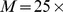. Note the apparent stretching along the 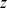-axis, which is discussed in the section *‘Implications for 3D reconstructions’*.

At high magnification, the DOC for the case of artificial tracers is relatively small. This permits a reconstruction of the three-dimensional flow field by collecting a series of measurements at several 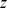-locations; here we use a 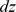 of 12 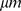. Figure 10 (A) shows a contour plot of the velocity magnitude at a single 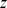 location (left-hand side). Along the white line, a slice in the 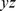 plane is extracted (top right). The dashed contour indicates the approximate blood vessel wall location, the dot represents the center. Both are based on a two-dimensional polynomial fit. The bottom right shows two velocity profiles, extracted along either the 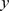 or 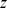 direction with respect to the center of the blood vessel; note how these represent ‘in-plane’ and ‘out-of-plane’ profiles with respect to the individual measurement planes (i.e. constant 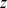) and thus also have a different spatial resolution. As is clear from the bottom right figure, the two profiles collapse, indicating a radially symmetric velocity profile.

For red blood cells, the DOC is much larger. If the same exercise is repeated as outline above, we obtain figure 10 (B). Note that due to practical limitations, it was not feasible to obtain two scans using the two modalities with the same embryo; the requirements of a single 3D scan are 20–30 times that of the individual measurements reported in the direct comparison experiments earlier in the manuscript. Again, the left-hand figure shows a single velocity magnitude contour plot. The top right shows a cross-section at the location indicated by the white line in the left-hand figure. The bottom right figure shows the two profiles extracted along the two orthogonal directions. In the two right-hand figures, we see the drawback of using red blood cells: the data suffers from very strong averaging along the 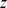 axis. This is evident from the two velocity profiles: the in-plane velocity is more ‘compact’ than the out-of-plane velocity profile. The profile along the 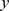 axis suggests a diameter that matches the one determined from visual inspection of the raw images. The 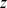 profile is stretched. Velocity results are nearly independent of 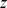 position, until one moves too far away from the centerline, where suddenly no meaningful results can be obtained (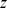 -50 or 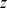 50). Note that a similar result was also observed by the pioneering study of Baker and Wayland [33]. At the core, their two-slit method is closely related to the present method, which can be seen as an evolution of this optical, correlation-based method.

### Conclusions and Outlook

In this study, we presented the first direct comparison of blood flow measurements using both artificial tracer particles and naturally-present particles - in this case red blood cells. The results are obtained in chicken vitelline veins and arteries, but they can readily be translated to other applications, provided that e.g. the dimensions of red blood cells and spatial dimensions are in a comparable range.

The main outcome of this study is that for relatively low magnifications, the results are nearly identical. This is caused by the fact that the depth-of-correlation, i.e. the depth over which data is averaged, is relatively large compared to the blood vessel diameter. In this ‘depth-saturated’ regime, both methods give a similar underestimation of the centerline velocity (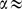
2/3). This means that experiments reported in the literature that are in this regime can be compared directly, regardless of the choice of tracer. Most studies do not explicitly mention what velocities are measured (e.g. centerline, depth-averaged), but the data can easily be corrected if sufficient specifics about the methodology are mentioned, in particular NA or magnification. Another implication is that there is no need for tracers in this regime, which greatly simplifies the experimental protocols. This is especially the case for repeated measurements for e.g. longitudinal studies [36] or for studies in very early developmental stages, where it is not feasible to inject tracer material.

For higher magnification (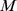 = 25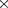), the difference between depth-of-correlation becomes more significant. In this case, the measurements obtained with artificial tracers underestimate the flow significantly less than those using red blood cells. The differences can be up to 50% of the real centerline velocity. Comparisons of literature results in this regime thus need to be done with great care.

The outcome of the present experimental study is supported by an ‘in silico’ PIV model, which can predict the averaging behaviour. It is based on a series of tracer images at different 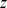 positions and some general assumptions of the flow field under consideration. The model takes into account the particular imaging characteristics of the microscope, illumination and tracer material. This relatively straightforward model can predict whether measurements are in the depth-saturated regime. For the present conditions, the transition is around 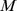 = 15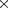 (see also Appendix S1), but it is impossible to give exact guidelines for other facilities. Fortunately, the procedure and model are relatively simple and in future studies the regime can be assessed fairly easily. The averaging in the 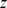 direction also limits the possibilities of obtaining three-dimensional velocity data using RBCs. The observed differences in in-plane and out-of-plane radial profiles also further support the conceptual measurement model based on the comparison measurements. For three-dimensional measurements, small artificial tracers will be preferable. As velocity measurements are also being used to reconstruct the wall geometry (by making use of the fact that the velocity approaches zero at the wall), these reconstructions will suffer from stretching in the z direction as well.

For very high magnifications (not considered here), the DOC for both cases will decrease. However, the physical size of red blood cells may inherently introduce averaging along the 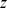 axis. Similarly, the in-plane resolution will be affected by the relatively large size of the RBCs. Therefore, in these measurements artificial tracers are still preferred. These measurements will also shed light on the hemodynamic environment near the vessel wall, in particular in the cell-free layer. Here, significant differences are naturally to be expected for artificial tracers and RBCs. These experiments require a refinement of the present facility (in particular regarding image stability and camera noise level), which is planned for future work.

## Supporting Information

Figure S1
**In silico PIV model: acquiring particle images and their cross-correlation function.** (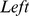) Raw image of 1.28 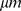 tracer particles taken at 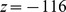 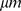, which here coincides with the focal plane. (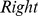) Autocorrelation of the particle images (inverted). NB: not to scale.(EPS)

Figure S2
**In silico PIV model: shifting the autocorrelation using known velocity profile.** 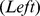 Stack of autocorrelation functions. 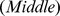 Velocity profile. 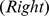 Displaced correlation functions. The dashed lines marked by ‘A’ and ‘B’ are also shown in figure 13 and represent contributions from in-focus (centerline, B) and out-of-focus (near wall, A) particle images.(EPS)

Figure S3 **In silico PIV model: summation of the correlation function to predict underestimation.** Individual displaced correlation functions and their sum (arbitrarily scaled in the vertical direction). Two individual contributions are labeled as ‘A’ and ‘B’, representing out-of-focus (near wall) and in-focus (centerline) results, respectively.
(EPS)

Figure S4 **Prediction for the underestimation based on the ‘in silico’ PIV model.** Reference data from Kloosterman et al. [22]. The inset shows the predictions for the old spatial averaging model.
(EPS)

Appendix S1 **In silico model for flow underestimation by micro-PIV.**
(PDF)
