# Supplementary material for: Accurate Blood Flow Measurements: Are Artificial Tracers Necessary?
Source: PLoS One. 2012 Sep 20;7(9):e45247. doi: 10.1371/journal.pone.0045247 (PMC3447936; doi:10.1371/journal.pone.0045247)
Supplement: Appendix S1 — In silico model for flow underestimation by micro-PIV. (PDF) [file pone.0045247.s005.pdf]

Accurate blood flow measurements: are artificial tracers necessary?

Christian Poelma[1,*], Astrid Kloosterman[1], Beerend P. Hierck[2], Jerry Westerweel[1]
1 Laboratory for Aero & Hydrodynamics, Delft University of Technology, Leeghwaterstraat 21, 2628 CA Delft, The Netherlands;
2 Department of Anatomy & Embryology, Leiden University Medical Center, PO Box 9600, 2300 RC Leiden, The Netherlands
∗ E-mail: c.poelma@tudelft.nl

Appendix S1: In silico model for flow underestimation by micro-PIV

In this appendix, we present an efficient method to predict the underestimation of the velocity field for a particular measurement facility and application. In the conventional 'synthetic PIV' method, image pairs are created based on a random distribution of idealized, Gaussian particle images, essentially trying to simulate the entire measurement process [1]. Here, we present a pragmatic, simpler method that avoids many of the assumptions required in synthetic PIV models. The model uses a similar approach as the ones introduced recently by Kloosterman et al. [2], Nguyen et al. [3] and Fouras et al. [4, 5]. These papers are also highly recommended for an in-depth discussion of the effects of (depth) averaging on the correlation procedure. The main difference with these methods is that they rely on fitting a model to particles images at different z-locations, while we can here avoid this step. We do this by utilizing the autocorrelation of real particle images and hereby by-pass most of the modeling steps (particle image approximation, synthetic image generation, cross-correlation). We describe the process step-by-step:

1. A droplet of a dilute solution containing tracer particles (either artificial tracers or red blood cells) is placed on a microscope slide and covered with a cover glass. Images are recorded using the same imaging modality (illumination, optics, camera setting, etc.) as used for the actual velocity measurements. Using a translation stage (motorized z-stage of the microscope or manually using an accurate translation stage), images are recorded at several z positions. Here, we used a separation between planes of dz of 10 and 12.5 μm, capturing images of 'out-of-focus' particles (above focal plane) and progressing through the focal plane, ending when the particles are again out-of-focus (below the focal plane). In figure S1 ($left$), an example of a raw image is shown, here taken at the focal plane (here $z = -166$ μm).

2. From each of the images, the auto-correlation is calculated. This gives an 'average' particle image, enlarged (by a factor $\sqrt{2}$ for a Gaussian particle image) due to the autocorrelation step. See figure S1 ($right$) for an example. Note that in a real experiment, particles images from subsequent frames are cross-correlated. The auto-correlation performed here can be interpreted as a measurement of a non-moving flow.

3. Assuming that the particle images are more or less radially symmetric and distributed randomly, the autocorrelation is also radially symmetric. Therefore, only the correlation as a function of radial position (r) needs to be stored for each z-plane.

4. A stack of autocorrelations is generated by interpolating the results from the previous steps to a finer resolution (here 1 μm), as shown in figure S2 ($left$): each horizontal line represents the autocorrelation of particle images at that particular z position. We here only show the part below the focal plane; note that in practice the focusing behaviour is often asymmetric with respect to the focal plane [6].

5. A parabolic flow profile is assumed (figure S2 (*middle*)). Here, we need to choose a particular spatial dimension of the flow geometry (e.g. blood vessel size). Also, we specify the maximum displacement (i.e. centerline velocity), which has a minor influence. The autocorrelation stack is displaced with the local value of the velocity profile, see figure S2 (*right*). This follows from the basic principle in PIV that a uniform displacement of particles leads to a single peak in the correlation result, representing the most likely displacement [7]. For instance, here the autocorrelation at $z = -116\ \mu m$ is displaced by 15 pixels to the right (see dashed line 'B').

6. The 'observed' correlation result will be the sum of the contributions at all z positions, so we sum all the 'displaced' correlation functions (see Figure S3). The maximum of the summed correlation will be the most likely displacement, i.e. the velocity that will follow from a PIV analysis. Two individual contributions are indicated by 'A' and 'B', representing a contribution from near-wall and centerline particles, respectively (see also Figure S2, right). Notice that 'B' represents the centerline displacement (dx_c), while the PIV algorithm will use the sum of all contributions (dx_m). The latter will yield a smaller displacement than the centerline displacement. As customary, the location of the maximum is refined by using a three-point Gaussian fit [7].

Notice that rather than displacing the actual particles (which is the case in a PIV experiment), we here displace the correlation result. This is allowed due to the linear nature of the correlation operator. The major assumption in this model is that the measurement result can be split into contributions from separate z planes, where in each plane the particles are moving at the local velocity (see also Fouras et al. [5]). The recording of single layers of tracer particles (step 1) is the physical manifestation of this assumption. This approach is allowed only for relatively sparse images, i.e. for low tracer concentrations. In this case particle images do not overlap and 'underlying' particles (at lower z positions) are not obscured by tracer particles closer to the sensor.

In figure S4, we show the predictions based on this 'in silico' model pertinent to the present study. The figure shows the (predicted) measurement result normalized with the real centerline velocity for a range of blood vessel diameters. The different lines represent 6 different cases: three magnifications ($M =$ 12.5, 15 and 25$\times$) for the two relevant tracer particle sizes (1.28 μm artificial tracer particles and 8 μm red blood cells). For the $M =$15 and 25$\times$ cases, reference data is available from measurements in glass capillaries [2], which is shown as the squares. The error bars represent the experimental uncertainty in those measurements. The new model predicts the observed underestimation reasonably well. The most important result from these predictions is the fact that for decreasing magnifications the underestimation converges to a constant value (0.65-0.7), and is no longer a value of magnification and/or blood vessel diameter (the former was also verified by other simulations; not shown). Minor variations are found for different tracer particle sizes and centerline displacements, which are caused by the interplay between correlation function shape and particle image [8].

For comparison, we also present the predictions of the underestimation using the spatial averaging model (equation 1) and theoretical predictions of the depth-of-correlation (table 1) in figure S4 (*Inset*) for the cases $M = 12.5$ and $M = 25$. The horizontal dashed line indicates the bottom limit of underestimation ($\alpha = V_{measured}/V_{centerline} = 2/3$): in this case the correlation depth is larger than the blood vessel diameter. Depending on the imaging method, velocities below this limit can be found. For instance, if non-moving tissue outside the blood vessel is captured in the image, this further biases the velocity to lower values. For the case with fluorescent tracer particles, this is not an issue.

Note how the two models provide contrary results: the results for artificial tracer and RBCs are closer to each other at $M = 12.5\times$ than at $M = 25\times$ for the new model. For the old spatial averaging model,

the opposite is the case. Furthermore, the differences are larger for large diameters for the new model, while they are *smaller* in the old spatial averaging model.

References

1. Lecordier B, Demare D, Vervisch L, Réveillon J, Trinité M (2001) Estimation of the accuracy of PIV treatments for turbulent flow studies by direct numerical simulation of multi-phase flow. Measurement Science and Technology 12: 1382.

2. Kloosterman A, Poelma C, Westerweel J (2011) Flow rate estimation in large depth-of-field micro-PIV. Experiments in Fluids 50: 1587-1599.

3. Nguyen C, Carberry J, Fouras A (2011) Volumetric-correlation PIV to measure particle concentration and velocity of microflows. Experiments in Fluids : 1–15.

4. Fouras A, Dusting J, Lewis R, Hourigan K (2007) Three-dimensional synchrotron x-ray particle image velocimetry. Journal of Applied Physics 102: 064916.

5. Fouras A, Lo Jacono D, Nguyen C, Hourigan K (2009) Volumetric correlation PIV: a new technique for 3D velocity vector field measurement. Experiments in fluids 47: 569–577.

6. Rossi M, Segura R, Cierpka C, Kähler C (2012) On the effect of particle image intensity and image preprocessing on the depth of correlation in micro-PIV. Experiments in Fluids 52: 1063-1075.

7. Adrian R, Westerweel J (2010) Particle Image Velocimetry. United Kingdom: Cambridge University Press.

8. Poelma C, Westerweel J (2011) Generalized displacement estimation for averages of non-stationary flows. Experiments in fluids 50: 1421-1427.

Supporting Information Legends

Figure S1. In silico PIV model: acquiring particle images and their cross-correlation function. (*Left*) Raw image of 1.28 μm tracer particles taken at $z = -116$ μm, which here coincides with the focal plane. (*Right*) Autocorrelation of the particle images (inverted). NB: not to scale.

Figure S2. In silico PIV model: shifting the autocorrelation using known velocity profile. (*Left*) Stack of autocorrelation functions. (*Middle*) Velocity profile. (*Right*) Displaced correlation functions. The dashed lines marked by 'A' and 'B' are also shown in figure S3 and represent contributions from in-focus (centerline, B) and out-of-focus (near wall, A) particle images.

Figure S3. In silico PIV model: summation of the correlation function to predict underestimation. Individual displaced correlation functions and their sum (arbitrarily scaled in the vertical direction). Two individual contributions are labeled as 'A' and 'B', representing out-of-focus (near wall) and in-focus (centerline) results, respectively.

Figure S4. Prediction for the underestimation based on the 'in silico' PIV model. Reference data from Kloosterman et al. [2]. The inset shows the predictions for the old spatial averaging model.
